# Telephone‐Based Triage After Pediatric Tonsillectomy: A Retrospective Analysis

**DOI:** 10.1002/oto2.70274

**Published:** 2026-06-29

**Authors:** Alyssa C. Chapel, Neila Kline, Saudamini Lele, Felicity M. B. Lenes‐Voit, Romaine Johnson, Cynthia Wang

**Affiliations:** ^1^ Department of Otolaryngology, Division of Pediatric Otolaryngology University of Texas Southwestern Medical Center Dallas Texas USA; ^2^ LSU Health Shreveport, Department of Pediatric Otolaryngology Shreveport Louisiana USA

**Keywords:** pediatric otolaryngology, pediatric tonsillectomy, quality, tonsillectomy

## Abstract

**Objective:**

To describe posttonsillectomy telephone triage utilization and short‐term outcomes in a single tertiary pediatric system.

**Study Design:**

Retrospective cohort study.

**Setting:**

Tertiary care children's hospital.

**Methods:**

All patients aged 0 to 18 years (N = 12,167) who underwent tonsillectomy between January 2020 and December 2024 were included. Primary outcomes included timing and type of telephone calls, emergency department (ED) visits, and bleeding events within 30 postoperative days. Secondary outcomes included intervention rates based on presentation pathway (telephone triage vs direct ED presentation).

**Results:**

Among 12,167 patients, 2483 (20.4%) contacted telephone triage within 30 days. Of callers, 2273 (91.5%) were managed without ED referral; 210 (8.5%) were referred to the emergency department. Of bleeding related calls, 111 (66.9%) were managed without ED referral, and 41/60 (68.3%) patients referred to the ED did not require surgical intervention. Telephone triage demonstrated 91.5% effectiveness and prevented 2273 ED visits. Patients utilizing telephone‐based triage were more likely to have Medicaid insurance (*P* < .001) and live in high‐disadvantage neighborhoods (*P* < .001).

**Conclusions and Relevance:**

Telephone‐based triage is an effective measure to help reduce ED visits for pediatric posttonsillectomy patients. The majority of patients who utilized telephone‐based triage were successfully managed at home across all types of complications. There is a potential $158,000 to $360,000 in healthcare savings seen after implementing this program. High‐disadvantage patients were more likely to use telephone‐based triage but achieved similar outcomes to low‐disadvantaged peers. In this cohort, a structured phone‐triage pathway safely managed most concerns without ED referral.

Pediatric tonsillectomy is a procedure performed to treat obstructive sleep apnea (OSA), recurrent tonsillitis or recurrent peritonsillar abscesses.^1^ In 2019, an estimated 559,900 ambulatory tonsillectomies and 7100 inpatient tonsillectomies were performed, making this one of the most common pediatric procedures in the United States.^2^ Inpatient tonsillectomy is recommended for patients under the age of 3, those with severe sleep apnea (apnea hypopnea index >10 events/h or O_2_ nadir <80%), those with BMI >95% without a sleep study, and patients with complex medical histories (ie, Down syndrome, neuromuscular disorder, craniofacial anomalies).^1^ Risks following tonsillectomy include hemorrhage (defined as primary <24 hours postoperatively or secondary if >24 hours postoperatively), severe pain, dehydration, and respiratory failure, and in rare instances death.^3^, ^4^, ^5^

With most pediatric tonsillectomy being performed on an outpatient basis, the majority of patients seeking care for complications present to the emergency department (ED) for treatment. Studies estimate ED revisit rates to be between 7.6% and 12.8% after pediatric tonsillectomy.^6^, ^7^, ^8^, ^9^ The average cost of an ED revisit following tonsillectomy is $1420.^10^ In efforts to reduce excess healthcare utilization following tonsillectomy, proposals for pretonsillectomy education or standardized telephone follow‐up have been implemented at different institutions. One study analyzing a pretonsillectomy caregiver education program found no significant difference in ED utilization following implementation.^11^ A randomized controlled trial studying implementation of a nurse telephone follow‐up postoperatively demonstrated lower healthcare service utilization for patients in the intervention group.^12^

This study aimed to describe timing and type of telephone calls, ED visits, and bleeding events within 30 days after pediatric tonsillectomy. Secondary outcomes included intervention rates based on presentation pathway (telephone triage vs direct ED presentation). We hypothesize that time‐based telephone triage reduces ED utilization following tonsillectomy.

## Methods

This retrospective cohort study was conducted at Children's Medical Center Dallas, a tertiary care hospital for children in the Dallas‐Fort Worth and North Texas areas. Approval came from the University of Texas Southwestern Medical Center IRB with a waiver of informed consent (IRB # STU‐2023‐0260). We reviewed all children aged 0 to 18 who had a tonsillectomy with or without adenoidectomy performed between January 2020 and December 2024 at Children's Medical Center Dallas. We pulled data from the hospital's internal Pediatric Tonsillectomy Registry and combined it with institutional data from the Pediatric Health Information System (PHIS) As a participating PHIS institution, Children's Medical Center Dallas data were linked to the Pediatric Tonsillectomy Registry using an institutional decryption key, enabling integration of PHIS‐derived structured variables—including cost data, clinical flags, and the Child Opportunity Index—to enhance data consistency and support comparability with established PHIS definitions. This study was conducted exclusively within our institution and does not compare outcomes across PHIS‐participating hospitals. Our hospital uses a telephone triage system, staffed 24/7 by trained nurses. Nurses follow a structured ABCDF protocol (Airway, Bleeding, Complications, Dysphagia, Fever) when parents call after their child has undergone a tonsillectomy; the threshold for ED referral for active bleeding is defined as greater than approximately 2 tablespoons of bright red blood. All calls are documented in Epic and recorded for quality review. The tonsillectomy registry logs calls every night at midnight. All call details can be reviewed if needed and are reviewed quarterly. We included patients who had a tonsillectomy. We excluded patients who did not have a tonsillectomy or had it outside our study period.

We grouped care pathways based on when and how patients contacted us: no contact (no phone call or emergency department visit), triage‐managed (a call only, no emergency referral), triage‐to‐ED (a call that led to an emergency referral), and direct ED (emergency visit with no call first). This categorization allows us to test our central hypothesis that the mode of contact reflects the efficacy and necessity of hospital resources posttonsillectomy. The primary outcomes measured were the use of telephone triage, emergency visits, hospital readmissions, and return to surgery for bleeding control within 30 days after surgery.

We defined 3 time periods after surgery: early (Days 2‐4), mid (Days 5‐7), and late (>7 days), based on when complications usually happen. We grouped call reasons as related to bleeding, pain, or vomiting/nausea, based on documentation from the telephone calls.

For bleeding problems, we assessed whether triage decisions aligned with the actual outcomes. Triage was appropriate if nurses managed bleeding calls safely by phone when no ED visit or surgery was needed, or referred patients when surgery was necessary. Triage was inappropriate if too many patients were sent to the ED with no intervention, or if patients went to the ED on their own for bleeding but did not need a procedure.

We combined patient ZIP codes with US Census Bureau coordinates to calculate straight‐line distances from home to the hospital (ZIP code 75207) using the Haversine formula. We then sorted these distances into 4 groups: ≤10 miles, 11 to 25 miles, 26 to 50 miles, and >50 miles.

We assessed neighborhood disadvantage using the Child Opportunity Index 2.0, which examines education, health/environment, and social/economic factors at the census tract level. We set thresholds: a disadvantage was defined as a health *z*‐score below −0.5, education *z*‐score below −0.5, and socioeconomic *z*‐score below −1.0. Each patient received a composite score ranging from 0 to 3, based on the number of disadvantaged domains, with a “high disadvantage” score being 2 or more domains.

To study confounders, we compared patient traits by their use of telephone triage. We used *t*‐tests for numerical data and chi‐square tests for categorical data. We analyzed time patterns with descriptive statistics and cross‐tabulations. We examined pathway use and triage accuracy by neighborhood disadvantage and distance, using chi‐square tests and column percentages. We then used logistic regression to determine whether disadvantage and distance affected triage use after controlling for clinical factors, in line with the relationships outlined in our directed acyclic graph. We set *P* < .05 as significant. All analyses used Stata 19. Missing data were handled using listwise deletion.

## Results

Of 12,471 pediatric patients who underwent tonsillectomy, 11,968 (95.97%) had complete PHIS data. Telephone follow‐up data were available for 12,066 (96.75%). The median age was 6.6 years (IQR 4.0‐9.0), and 5775 (46.3%) were female. Geographic distance data were available for 12,039 (96.5%) patients. The median distance from the hospital was 16.6 miles (IQR 10.0‐28.0).

Patient characteristics differed significantly between telephone triage users and nonusers (Table 1). Patients using telephone triage were slightly older (7.2 vs 7.0 years, *P* = .003). They were more likely to have Medicaid insurance (73.8% vs 56%, *P* < .001), and more likely to live in high‐disadvantage neighborhoods (39.8% vs 26.3%, *P* < .001). Clinical comorbidities, including OSA, obesity, and complex chronic conditions, were similar between groups.

**Table 1:** Patient Characteristics by Telephone Triage Utilization

	No telephone call	Telephone call	Total	*P*‐value
	n = 9684	n = 2483	N = 12,167	
Age at surgery, years				.003
Mean (SD)	7.0 (3.6)	7.2 (4.0)	7.0 (3.7)	
Sex, n (%)				.263
Female	4278 (44.2%)	1318 (53.1%)	5596 (46.0%)	
Male	4981 (51.4%)	1462 (58.9%)	6443 (53.0%)	
Race/Ethnicity, n (%)				.038
Hispanic	3944 (40.7%)	1218 (49.0%)	5162 (42.4%)	
Non‐Hispanic Black	1834 (19.0%)	588 (23.7%)	2422 (19.9%)	
Non‐Hispanic White	2557 (26.4%)	723 (29.1%)	3280 (27.0%)	
Other	403 (4.2%)	110 (4.4%)	513 (4.2%)	
Insurance, n (%)				<.001
Commercial	2907 (30.0%)	666 (26.8%)	3573 (29.4%)	
Medicaid	5426 (56.0%)	1833 (73.8%)	7259 (59.7%)	
Government: Other	181 (1.9%)	56 (2.3%)	237 (1.9%)	
Neighborhood disadvantage score, n (%)				<.001
No disadvantage	4411 (45.5%)	1119 (45.1%)	5530 (45.5%)	
Single domain	2305 (23.8%)	672 (27.1%)	2977 (24.5%)	
Two domain	1892 (19.5%)	757 (30.5%)	2649 (21.8%)	
Multi‐domain	651 (6.7%)	232 (9.3%)	883 (7.3%)	
High neighborhood disadvantage (≥2 domains), n (%)				<.001
No	6716 (69.4%)	1791 (72.1%)	8507 (70.0%)	
Yes	2543 (26.3%)	989 (39.8%)	3532 (29.0%)	
Distance from hospital, n (%)				<.001
≤10 miles	2256 (23.3%)	792 (31.9%)	3048 (25.1%)	
11‐25 miles	4249 (43.9%)	1273 (51.3%)	5522 (45.4%)	
26‐50 miles	2194 (22.7%)	525 (21.1%)	2719 (22.4%)	
>50 miles	560 (5.8%)	190 (7.7%)	750 (6.2%)	
Patient risk category, n (%)				<.001
High risk	2560 (26.4%)	967 (38.9%)	3527 (29.0%)	
Low risk	3079 (31.8%)	787 (31.7%)	3866 (31.8%)	
OSA	3620 (37.4%)	1026 (41.3%)	4646 (38.2%)	

Continuous variables presented as mean (SD); categorical variables as n (%). *P*‐values from linear regression (continuous) and Pearson chi‐square (categorical).

Abbreviations: OSA, obstructive sleep apnea; SD, standard deviation.

Of 12,167 patients with complete pathway data, 9225 (75.8%) had no postoperative contact (Table 2). Another 2273 (18.7%) were managed by telephone triage alone, with triage effectively preventing about 2273 potential ED visits, or roughly 1 in 5 overall postoperative pathways being resolved through telephone alone. Meanwhile, 210 (1.7%) required triage‐to‐ED referral, and 459 (3.8%) presented directly to the ED. Patients who utilized telephone triage had an ED utilization rate of 8.46%, compared to a rate of 4.74% for nonusers. Telephone triage demonstrated 91.5% effectiveness (2273/2483 callers managed without ED referral), underscoring its potential to reduce strain on emergency services.

**Table 2:** Care Pathway Distribution and Temporal Patterns

Panel A: Care pathway distribution (N = 12,167)		
Care pathway	N	Percentage
No contact	9225	75.8%
Triage‐managed	2273	18.7%
Triage → ED	210	1.7%
Direct ED	459	3.8%

Panel B: Temporal distribution of complications by phase (N = 2483 callers)				
Complication type	Early phase (Days 2‐4)	Mid phase (Days 5‐7)	Late phase (>7 Days)	Total N
Bleeding calls	39 (25.7%)	72 (47.4%)	41 (27.0%)	152^a^
Pain calls	313 (41.6%)	363 (48.2%)	77 (10.2%)	753
Vomiting/nausea calls	52 (67.5%)	17 (22.1%)	8 (10.4%)	77

Panel C: Timing summary statistics (N = 12,167)		
Outcome	Median (Days)	IQR
Days to phone call	5.0	3.0‐7.0
Days to ED visit	6.0	3.0‐7.0

Care pathway utilization and temporal patterns of posttonsillectomy complications among 12,167 patients. Panel A shows overall pathway distribution. Panel B demonstrates temporal clustering by complication type. Panel C summarizes healthcare contact timing.

^a^ Bleeding call total reflects calls with documented phase timing (n = 152 of 166 total bleeding calls).

Telephone triage occurred at a median of 5 days postoperatively (IQR, 3‐7 days). ED visits occurred at a median of 6 days (IQR, 3‐7 days). Complications followed predictable patterns: vomiting‐related calls were concentrated early (67.5% on Days 2‐4). Bleeding calls peaked mid‐phase (47.1% on Days 5‐7). Pain calls occurred throughout, with a slight predominance during the mid‐phase (48.1% on Days 5‐7).

Among 166 patients with bleeding‐related telephone calls, 111 (66.9%) were managed conservatively without ED referral. Of 60 patients who went directly to the ED with bleeding complications, 41 (68.3%) did not require surgical intervention. Overall triage appropriateness for bleeding complications was 67.7% (111/164). This represented about 152 potentially avoidable ED visits (111 conservatively managed + 41 direct ED patients without intervention).

Distance to the hospital influenced the use of care pathways (*P* < .001) (Table 3). Patients living 11 to 25 miles from the hospital used telephone triage most often (45.8% of triage users). Only 6.8% of triage users lived more than 50 miles away. The use of direct ED decreased with distance, from 26.9% of direct ED patients living within 10 miles to just 2.6% living more than 50 miles from the hospital.

**Table 3:** Triage Effectiveness and Equity Analysis

Panel A: Triage utilization by social and geographic factors			
Factor	Low disadvantage	High disadvantage	*P*‐value
Telephone triage use	1791/8507 (21.1%)	989/3532 (28.0%)	<.001
Distance category	Triage utilization	Total N	
≤10 miles	792/3048 (26.0%)	3048	
11‐25 miles	1273/5522 (23.1%)	5522	
26‐50 miles	525/2719 (19.3%)	2719	
>50 miles	190/750 (25.3%)	750	

Panel B: Triage success rates (among callers)			
Factor	Triage‐managed	Triage → ED	Success rate
Low disadvantage	1459/1593 (91.6%)	134/1593 (8.4%)	91.6%
High disadvantage	810/885 (91.5%)	75/885 (8.5%)	91.5%
Overall	2273/2483 (91.5%)	210/2483 (8.5%)	91.5%

Panel C: Bleeding management equity				
Measure	Low disadvantage	High disadvantage	Overall	*P*‐value
Conservative management	73/110 (66.4%)	38/55 (69.1%)	111/166 (66.9%)	.715
Triage appropriateness	73/113 (64.6%)	38/51 (74.5%)	111/164 (67.7%)	.209

Panel D: Healthcare Utilization Impact	
Metric	Value
Triage success rate	91.5% (2273/2483)
Bleeding calls managed conservatively	66.9% (111/166)
Direct ED bleeding patients without intervention	68.3% (41/60)
Total triage‐managed (potentially avoidable ED visits)	2273

Equity analysis of telephone triage effectiveness across social determinants and geographic distance. All measures demonstrate equitable access and quality across disadvantage levels, with triage serving as an effective safety net for high‐disadvantage populations.

Patients from high‐disadvantage neighborhoods used telephone triage more frequently than those from low‐disadvantage areas (35.6% vs 27.5%, *P* < .001). They were less likely to go directly to the ED (27.6% vs 72.4% of direct presentations, *P* < .001). This trend was seen across the disadvantage spectrum. Triage use increased stepwise with disadvantage, from no disadvantage (39.7%) to multi‐domain disadvantage (8.1% of triage users vs 6.9% of nonusers).

Triage effectiveness remained equitable across all levels of disadvantage. Among telephone triage users, 91.5% were managed without an ED referral, regardless of neighborhood disadvantage (*P* = .998). For bleeding complications, triage appropriateness did not differ significantly by disadvantage level (67.7% overall, *P* = .209) or geographic distance (*P* = .540).

Logistic regression analysis confirmed that the link between neighborhood disadvantage and telephone triage use remained strong after adjusting for distance and clinical factors. The adjusted odds ratio for high disadvantage was 1.34 (95% CI 1.21‐1.48). In the unadjusted model, the estimate was 1.36 (95% CI, 1.25‐1.48), indicating minimal confounding by distance.

## Discussion

In this study, we describe the effect of telephone‐based triage on ED utilization for posttonsillectomy patients. Seventy‐five percent of patients did not use telephone‐based triage or the emergency department following their tonsillectomy. Most of the patients who utilized telephone‐based triage were managed over the phone and did not require emergency room evaluation. Overall, telephone triage demonstrated 91.5% effectiveness in management of posttonsillectomy complications and managed an estimated 2273 calls without ED referral. There were 166 telephone‐based triage encounters relating to bleeding, and of these bleeding‐related calls 111 (66.9%) were managed conservatively and did not require ED presentation. Within the cohort directly presenting to the emergency room, 60 visits were related to bleeding and 68.3% of these patients were managed conservatively. This suggests that telephone‐based triage has the potential to conservatively manage minor bleeding‐related complications as effectively as those who present to the emergency room.

Telephone‐based triage also shows potential to reduce healthcare costs. Using conservative estimates from the literature of $1420 per ED visit for post‐tonsillectomy complications, the 152 potentially avoidable ED visits identified in this study represent approximately $216,000 in cost savings.^13^ Our institutional data showed actual ED visit costs averaged $7442, though formal cost‐effectiveness analysis was beyond the scope of this study. These preliminary findings suggest telephone triage may provide economic value alongside clinical effectiveness and equity benefits, warranting future dedicated cost analyses.

Patients who utilized telephone‐based triage were more likely to have Medicaid insurance (*P* < .001) and more likely to live in a high‐disadvantage neighborhood (*P* < .001). Patients from high‐disadvantage neighborhoods were less likely to present directly to the ED for posttonsillectomy complications (*P* < .001). The odds ratio for telephone‐based triage use in high‐disadvantaged neighborhoods is 1.34 (96% CI 1.12‐1.48) and showed minimal difference when adjusted for distance and clinical factors. Telephone‐based triage for post‐tonsillectomy complications expands healthcare access for this population, and potentially alleviates burden associated with ED visit (ie, transportation, financial cost, time) by supporting a higher number of complications to be managed conservatively at home. Higher utilization of telephone‐based triage in the high‐disadvantage cohort could reflect the higher rates of post‐tonsillectomy complications previously established in this population.^14^, ^15^ There was no disparity in telephone‐based triage effectiveness based on neighborhood disadvantage (*P* = .209) or geographic distance (*P* = .540). Importantly, high‐disadvantaged patients utilizing telephone‐based triage achieved equivalent outcomes to low‐disadvantaged users.

This retrospective, single‐system analysis is subject to selection and misclassification biases. This was primarily a descriptive and quality improvement study without controls. We conducted a retrospective cohort study and therefore our data may be susceptible to selection bias. However, the higher ED utilization rate seen among telephone‐based triage users supports that higher‐risk patients utilizing this service appropriately self‐selected. Additionally, this was conducted at a single, tertiary care children's hospital in an urban area and may not be generalizable to other practice types (ie, private or rural settings). Patients may have sought care at outside EDs that were not captured, potentially undercounting ED use, particularly for families living farther from our hospital. However, use of nursing scripts and the reliable temporal patterns observed in this cohort make this a replicable protocol which could be instituted for different care settings. Future research could be conducted on a multi‐institutional level, or designed as a randomized control trial, to increase the power of the study.

## Conclusion

Tonsillectomy is one of the most common pediatric outpatient surgical procedures in the country, and while complication rates remain overall low there is increased ED utilization seen following this procedure. A telephone‐based triage was implemented at a single, tertiary‐care academic institution to help reduce ED use after tonsillectomy. Most patients had no contact with the triage center or ED following tonsillectomy. Of patients who used telephone‐based triage, the majority were effectively managed conservatively. Telephone‐based triage also proved to be effective for higher‐disadvantage patients, and there was no disparity in the use of this resource based on disadvantage. This study demonstrates that telephone‐based triage is an effective tool in the postoperative management of pediatric tonsillectomy patients and has potential to reduce ED utilization following this procedure.

## Disclaimer

This manuscript was edited with the assistance of ChatGPT, an AI language model developed by OpenAI, to improve clarity, conciseness, and grammar. The authors take full responsibility for the content and accuracy of the final version.

## Author Contributions

**Alyssa C. Chapel**, MD, corresponding author, data interpretation, manuscript drafting, manuscript review; **Neila Kline**, MD, manuscript drafting and review; **Saudamini Lele**, MD, manuscript review; **Felicity M. B. Lenes‐Voit**, MD, manuscript review; **Romaine Johnson**, MD, MPH, project formation, data collection, statistical work, data analysis, manuscript drafting, manuscript review; **Cynthia Wang**, MD, data analysis, manuscript drafting, manuscript review.

## Disclosures

### Competing interests

None.

### Funding source

None.
